# Clean Cellars

**Published:** 1875-06

**Authors:** 


### CLEAN CELLARS.

Cellars are very useful places when kept clean, and very dangerous adjuncts to a dwelling, when permitted to remain dirty. As they are the catch-all in the autumn and during the winter, it is important that they should be effectively cleaned in the spring.

The sun is the greatest purifier in the world, but it has little opportunity to exercise its powers in the underground rooms. So we must take extra pains to keep the cellar clean and sweet, or the health of the family will be likely to suffer. The air exposed to the decaying substances contained in very many cellars, is unfit to breathe; and the only security for the health of the occupants of the upper apartments, is in keeping the cellar pure, since the bad air below finds a multitude of channels of escape.

The first condition of healthfulness is secured when the cellar is cleaned of all its rubbish. This process must be very thorough in order to effect its object. Not a spoonful of loose dirt should be left anywhere. The top, sides and bottom of the excavation must be swept and brushed with long-handled or short-handled brooms, until no more dust or cobwebs can be found. The accumulated *debris* must be carried out and disposed of, in such a way that the emanations from it will do no harm. All tables, shelves, boxes, and barrels must be removed, and thoroughly scrubbed and exposed to the sun for some days before being carried back. The cellar should be opened as much as possible, in sunny days, so that it may get a frequent bath of pure air.

After the dirt has been removed, a little chemistry should be employed. The chemical effect desired is best secured by sifting air-slaked lime around, close to the walls and in out-of-the-way places, and afterwards giving the wood-work overhead, and the walls, a good coat of whitewash, made of fresh lime and boiling water, mixed to about the thickness of cream. It should be rubbed into the surfaces and is much better if applied hot.

Persons who keep milk or cream or butter in their cellars will secure them against taint, only by great care. The products of the dairy are greatly influenced by the air surrounding them, and by nothing more surely than by the odors and impurities which they themselves generate in decomposing.

A pan of sour, mouldy milk or cream, allowed to stand for days and weeks, will contaminate everything in the cellar, and impart a rank flavor to all articles of food, besides rendering them positively unwholesome. It is true economy to clean out all decomposing articles, instead of allowing them to remain, as some do, in the belief that they may be used by and bye. So also, if milk gets spilt in the cellar, it should be carefully wiped up, without delay.

Lime is a valuable purifier, and should be kept on hand for frequent use. A little of the powder which slakes off from the lumps should be sprinkled about the cellar from time to time. It is unfriendly to rats, as their feet suffer when they tread upon it. Its employment either dry or as a wash, cannot be too strongly urged upon all who desire light, clean, sweet and wholesome cellars.

